# Optimization and *In Vivo* Toxicity Evaluation of G4.5 Pamam Dendrimer-Risperidone Complexes

**DOI:** 10.1371/journal.pone.0090393

**Published:** 2014-02-28

**Authors:** Maria Jimena Prieto, Nahuel Eduardo del Rio Zabala, Cristian Hernán Marotta, Hector Carreño Gutierrez, Rosario Arévalo Arévalo, Nadia Silvia Chiaramoni, Silvia del Valle Alonso

**Affiliations:** 1 Biomembrane Laboratory, Department of Science and Technology, National University of Quilmes, Buenos Aires, Argentina; 2 IMBICE-CONICET, CCT La Plata, Argentina; 3 Department of Cell Biology and Pathology, Institute of Neuroscience of Castilla y Leon, School of Medicine, University of Salamanca, Salamanca, Spain; Brandeis University, United States of America

## Abstract

Risperidone is an approved antipsychotic drug belonging to the chemical class of benzisoxazole. This drug has low solubility in aqueous medium and poor bioavailability due to extensive first-pass metabolism and high protein binding (>90%). Since new strategies to improve efficient treatments are needed, we studied the efficiency of anionic G4.5 PAMAM dendrimers as nanocarriers for this therapeutic drug. To this end, we explored dendrimer-risperidone complexation dependence on solvent concentration, pH and molar relationship. The best dendrimer-risperidone incorporation (46 risperidone molecules per dendrimer) was achieved with a mixture of chloroform:methanol 50∶50 v/v solution pH 3. In addition, to explore the possible effects of this complex, *in vivo* studies were carried out in the zebrafish model. Changes in the development of dopaminergic neurons and motoneurons were studied using tyrosine hydroxylase and calretinin, respectively. Physiological changes were studied through histological sections stained with hematoxylin-eosin to observe possible morphological brain changes. The most significant changes were observed when larvae were treated with free risperidone, and no changes were observed when larvae were treated with the complex.

## Introduction

The antipsychotic drug risperidone, 3-[2-[4-(6-fluoro-1,2-benzisoxazol-3-yl)-1-piperidinyl]ethyl]-6,7,8,9-tetrahydro-2-methyl-4H-pyrido[1,2-*a*] pyrimidin-4-one (Risp), belongs to the chemical class of benzisoxazole and it is one of the drugs most widely used in the treatment for autism spectrum disorders (ASD) [1], [2]. ASD, which occur in 1 out of 150 individuals [2], include different neurodevelopment disorders that manifest mainly in the earlier years of life [3], affecting language, communication and reciprocal social interaction development [4]. Risp has low solubility in aqueous medium and, when orally administered, exhibits low bioavailability due to extensive first-pass metabolism and high protein binding (>90%) [5]. Moreover, non-targeted delivery usually results in numerous side effects. Since Risp target organ is the brain, it is necessary not only to develop a strategy to improve drug bioavailability, by avoiding first-pass metabolism, but also to achieve the desired drug concentration at the site of action, thus reducing undesirable side effects [1]. In the last years, strategies with chemical therapies, particularly the design of nano-structured drug carrier systems [6], have been proposed to overcome these issues regarding ASD treatment. However, these kinds of carriers (plain, ultradeformable, stealth, pH-sensitive liposomes, immunoliposomes, nanoparticles and dendrimers) must be carefully designed and/or chosen because their pharmacokinetics, biodistribution, and tissue selectivity depend exclusively on the nanocarrier structure [1], [7]–[9].

In this sense, dendrimers are exceptional polymers presenting important advantages over conventional linear or branched ones such as polyethylene terephthalate or comb polymers, respectively [10],[11]. These advantages include monodispersity [12], controlled size in the range of nanometers, controlled number of surface groups, and extremely high area/volume ratio. Only intermediate generation (3.5–5 G) dendrimers are suitable drug carriers, with structures open enough to enable the loading and subsequent release of molecules in a controlled fashion [13]–[15]. Since, in the last years, PAMAM dendrimers have been found to be useful to improve the solubility of low aqueous soluble drugs [16], [17], the present work aims to enhance Risp solubility by means of PAMAM dendrimers. On the other hand, we used the zebrafish (*Danio rerio*) as an ideal model to study developmental neurobiology and other fields of biomedicine. The zebrafish is a teleost of the Cyprinid family, with several advantageous features for use in the laboratory: its small size (no more than 5 cm in adults) allows easy maintenance of several individuals with relatively low costs; females lay a large number of eggs; embryos develop rapidly and are semitransparent 24 hours post-fertilization (hpf); and embryos have a sequenced genome and numerous mutant and transgenic lines [18]–[22].

**Figure 1:**
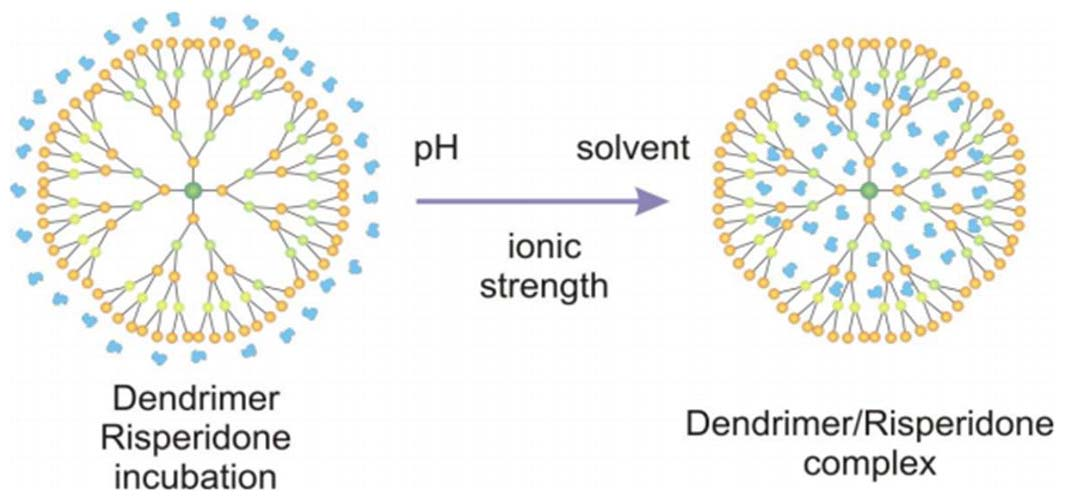
Dendrimer-Risperidone complex. Scheme of Risp complexation with PAMAM dendrimers Generation 4.5 (DG4.5) at different solvent, pH and molar relationship.

Thus, our proposal was the optimization of Risp complexation with PAMAM dendrimers Generation 4.5 (DG4.5) at different solvent concentrations, pH and molar relationship (Figure 1). In addition, we analyzed the *in vivo* effects of risperidone and DG4.5-Risp complexes on heart rate and brain development of zebrafish larvae.

## Materials and Methods

### Materials

Poly(amidoamine) (PAMAM) dendrimer G4.5 (–COOH) (molecular weight = 26,258 g/mol, 128 carboxyl end groups) (DG4.5) was purchased from Sigma–Aldrich, Argentina. Risperidone (Risp) 99.0% was donated by Janssen Cilag Laboratory, Argentina. All other reagents used were of analytical grade.

### Preparation of DG4.5-Risp Complex

**Figure 2:**
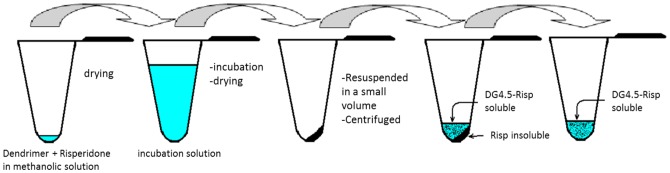
Preparation of DG4.5-Risp Complex. DG4.5 was combined with a specific amount of Risp in methanol solution and methanol was immediately evaporated. All incubations were carried out for 48×*g* for 10 min, in order to separate the DG4.5-Risp complexes (soluble Risp) from the non-incorporated Risp (insoluble).

DG4.5 was obtained as previously [9]. Briefly, DG4.5 was combined with a specific amount of Risp in methanol solution at 1∶100 and 1∶250 DG4.5:Risp molar ratios, and methanol was immediately evaporated in a Speed Vac SAVANT at 25°C for 15 min (1010 SAVANT). After evaporation, Risp and PAMAM DG4.5 were incubated with 1 ml of: a) chloroform:methanol 70∶30; b) chloroform:methanol 50∶50; c) chloroform:methanol 90∶10; d) chloroform:methanol 50∶50 pH 3; e) chloroform:methanol 50∶50 pH 6; f) chloroform:methanol 50∶50 pH 9; g) chloroform:methanol 50∶50 pH 3 with additional drying; h) chloroform:methanol 50∶50 pH 6 with additional drying; or i) chloroform:methanol 50∶50 pH 9 with additional drying. All incubations were carried out for 48 h at room temperature (20°C) with continuous stirring. Finally, solvents were completely evaporated in a Speed Vac SAVANT. The solid residues obtained were dissolved in 0.1 ml of phosphate buffer (PBS), at room temperature, and centrifuged at 10,000×*g* for 10 min, in order to separate the DG4.5-Risp complexes (DG4.5-Risp) (soluble Risp) from the non-incorporated Risp (insoluble) (Figure 2). Complex's pH was adjusted to physiological pH with phosphate buffer PBS 7.4. The drug does not precipitate as it is incorporated into dendrimers and dendrimers are water soluble.

If there were traces of MeOH and/or chloroform, they were determined prior to preparing the final solution complexes. Steps followed were: samples of each condition, in quintuplicate, were vacuum dried in a Speed Vac SAVANT 10010 until dryness. Two sets of samples were prepared in a parallel form. One set of samples was submitted to an additional drying procedure in an oven for 2 h at 40°C, the other set remained at room temperature, and was used as a control. Afterwards, all samples were suspended in the buffer solution and quantification of Risp was stated as in section 2.3. All samples achieved the same result for each condition between sample and control, confirming that the second step was unnecessary and the absence of solvent present was confirmed.

### Risperidone Quantification

**Figure 3:**
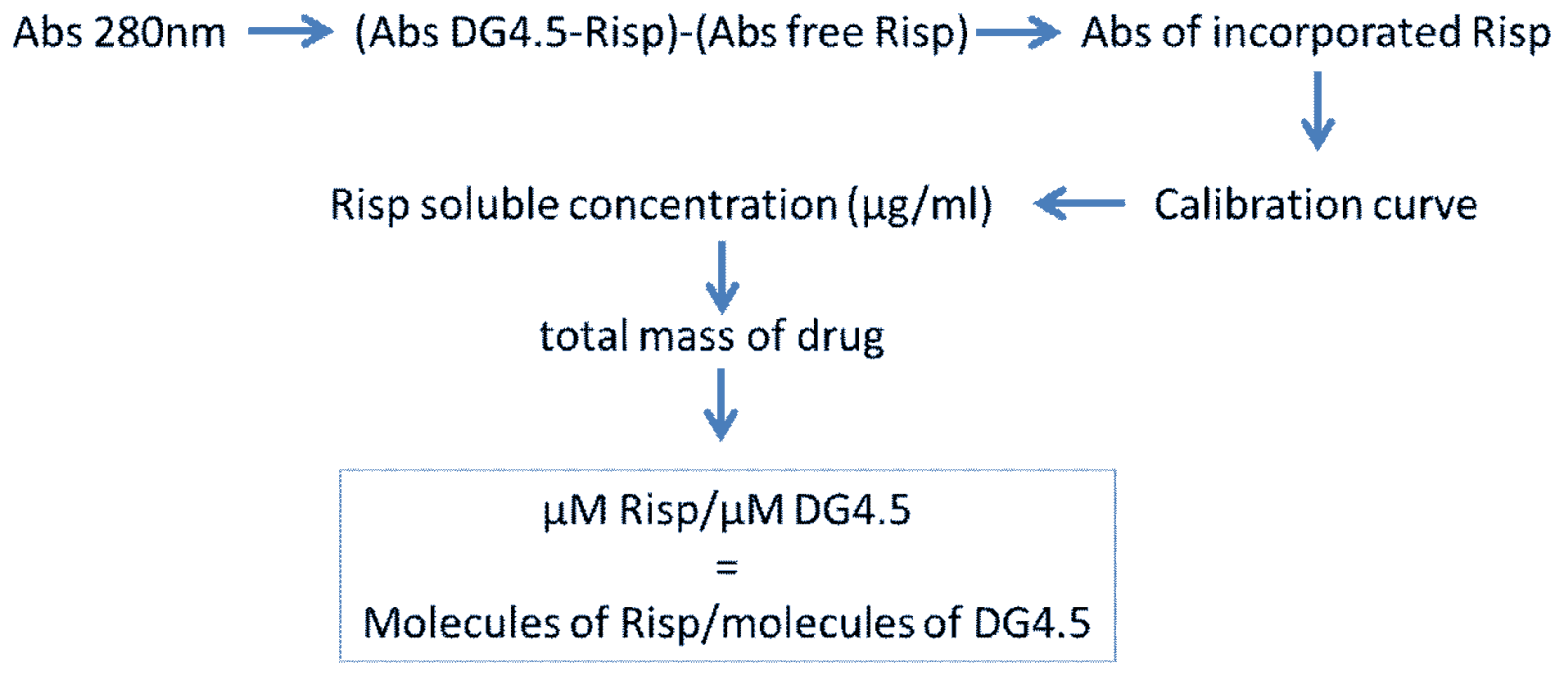
Risperidone quantification. Scheme shows the determination of number of Risp molecules per dendrimer. The amount of Risp was quantified by measuring the absorbance at 280–Vis spectrophotometer.

**Figure 4:**
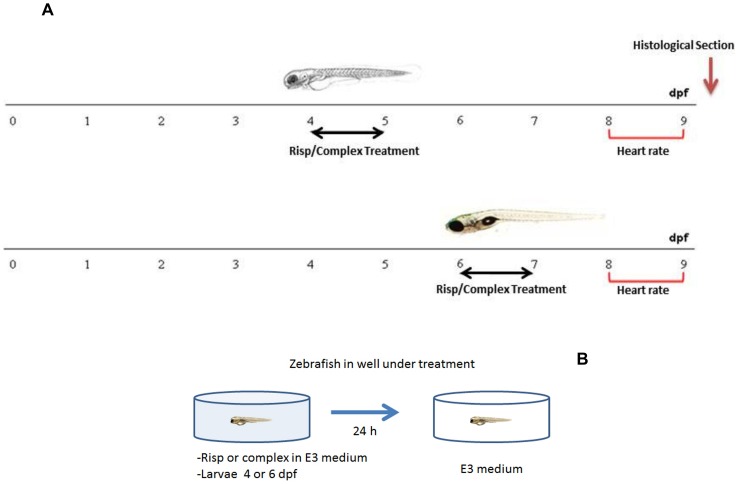
Timeline representing the stage specificity of the effects of risperidone and DG4.5-Risp in developing zebrafish. Larvae were exposed to 5 µM risperidone or DG4.5-Risp for 24-h periods from 4 dpf or 6 dpf and subsequently rescued into a conditioned E3 medium (A). Schematic representation of the in vivo treatment (B).

The amount of Risp was quantified by measuring the absorbance at 280 nm with a UV–Vis NanoDrop1000. The calibration curve of Risp in PBS was linear in a concentration range of 0.1–100 µg/ml (r^2^ = 0.99) (Figure 3) [23], [24]. DG4.5 does not absorb at this wavelength (see Figure 4).

**Figure 5:**
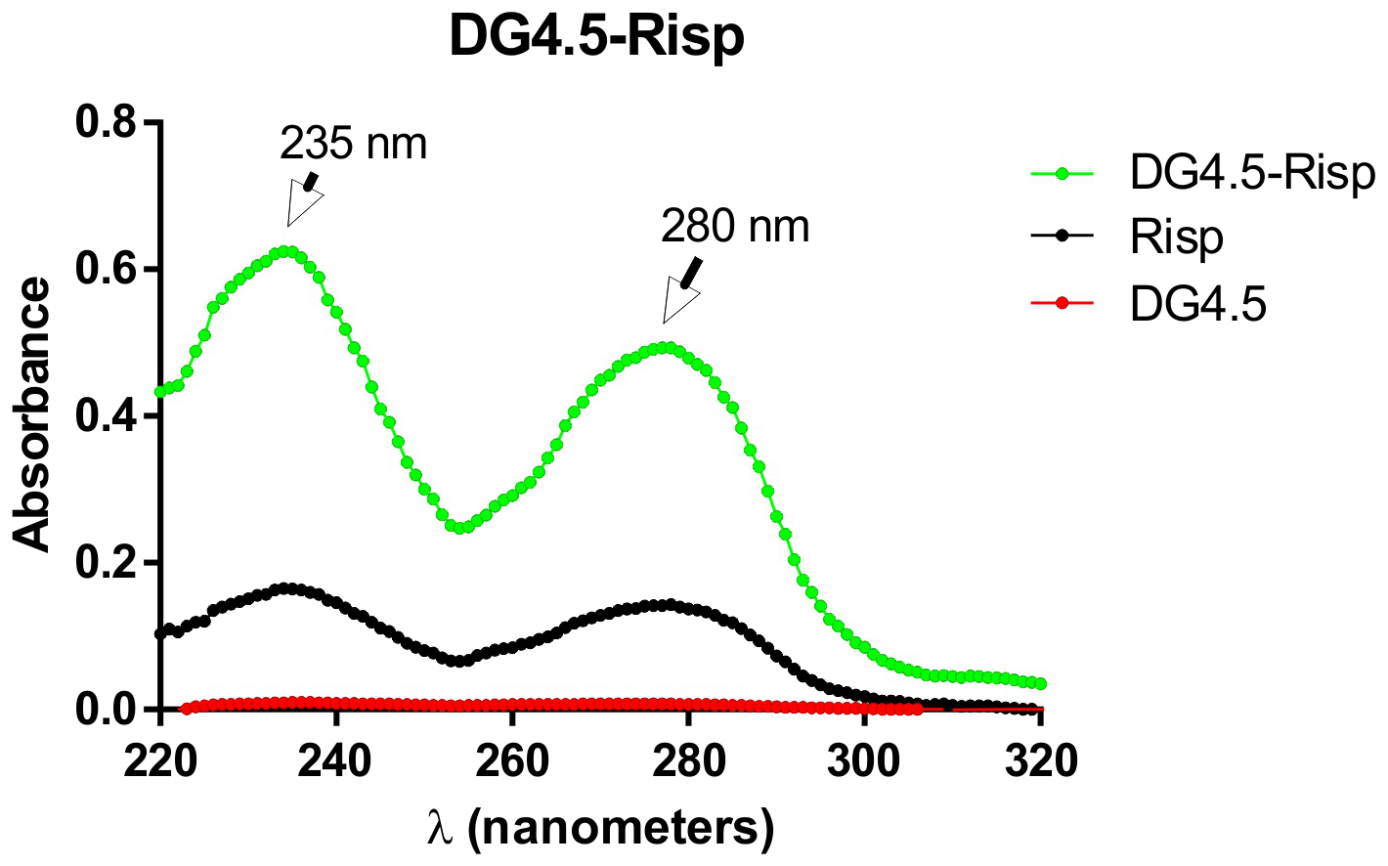
Evaluation of the Dendrimers-Risperidone complex formation. Absorbance Spectrum DG4.5-Risp (green) and free Risp (black) under experimental conditions determined as optimum.

From absorbance vs. wavelengths graphics at different concentrations like Figure 5, a double reciprocal plot of 1/absorbance versus 1/Risp concentration was calculated and linear regression was linear, and the binding constant (k) estimated from the ratio of the intersection to the slope was k = 4 [25], [26].

### *In Vitro* Release Studies

*In vitro* release of Risp from DG4.5-Risp complexes was studied in PBS by using a micro-dialysis eppendorf tube diffusion technique, by replacing the top internal flap-cover of a 0.5-ml eppendorf tube with a dialysis membrane. This technique was implemented and adapted to overcome micro-quantities of the released drug. DG4.5-Risp complexes were sealed into the micro dialysis eppendorf tube (MW cut-off: 12000 from Sigma-Aldrich, Argentina) and incubated in PBS under continuous stirring. The Risp release experimental design consisted of collecting aliquots at pre-determined time intervals from the incubation medium, and storing them at 4°C for quantitative analysis. Each aliquot withdrawn is replaced afterwards by an equal volume of fresh medium to maintain volume and to be considered within the calculus. On the other hand, pH and temperature are controlled to ensure they remain unchanged. The assay was repeated three times and the amount of released Risp was determined by absorbance at 280 nm, as described in Section 3.3. Data were analyzed with GraphPad Prism 5 *t-test*.

### Characterization of DG4.5-Risp Complexes

The spectra of the collected samples were characterized placing 1 ml of each of the residues into the attachment plate to measure attenuated total reflectance (ATR). The determinations were carried out in a spectrophotometer IRAffinity-1 Fourier Transform Infrared Compact Shimadzu. After 25 scans in the range of 650 cm^−1^ to 4000 cm^−1^, the spectrum was withdrawn with a resolution of 0.5 cm^−1^. The IR spectra were analyzed with solution software, version 1.50, supplied by the manufacturer.

Mean particle size and zeta potential of the complexes were determined by dynamic light scattering with a Nanozetasizer (Malvern Instruments, Malvern, Worcestershire, UK).

### *In Vivo* Studies: Animals

Adult zebrafish (*Danio rerio*) used as breeding individuals belong to the AB line, provided by the Department of Cell Biology and Pathology, University of Salamanca (Spain) for histological assays. The animals were kept in tanks at 28°C on a 14/10 h light/dark cycle as previously established [18]. In this study, embryos refer to zebrafish prior to hatching (0–3 dpf), while larvae refer to post-hatching animals (over 3 dpf). Embryos were obtained from natural mating, and all embryos/larvae used in these experiments were reared at 28.5°C on a 14/10 h light/dark cycle in conditioned E3 medium (NaCl 0.29 g/l, KCl 0.012 g, CaCl_2_ 0.036 g/l and MgSO_4_ 0.039 g/l in deionized water, and 50 ppb methylene blue (Panreac) to inhibit fungal growth).

### Ethics Statement

The animals were handled following the European Union directives (86/609/EEC and 2003/65/EC) and Spanish legislation (RD 1201/2005, BOE 252/34367-91, 2005). Full details of the study were approved by the Bioethics Committee of Salamanca University (CBE/30/07/08). The animals were anesthetized by a tricaine methanesulfonate solution and all efforts were made to minimize suffering.

### Embryo Collection

The evening before spawning, breeding pairs of specimens were transferred to rearing tanks. These tanks were kept at 28.5°C. The first light stimulus after the dark cycle induced egg lay. The eggs obtained were prepared in petri dishes in E3 medium. Only fertilized eggs in good condition were selected for further treatment; the others were discarded. The characteristics of eggs were determined with a stereomicroscope (Leica Zoom 2000).

### Exposure to Risperidone and PAMAM Complexes

Risperdal tablets (Janssen Cilag Laboratory, 2 mg Risp) were dissolved in E3 medium and prepared as a 0.5, 5 and 25 µM solution. The larvae were divided into four groups and then treated with i) Risp at 4 dpf for 24 h, ii) Risp at 6 dpf for 24 h, iii) DG4.5-Risp at 4 dpf for 24 h, and iv) DG4.5-Risp at 6 dpf for 24 h, or v) medium (controls). Larvae were exposed to 5 µM Risp for 24-h periods and subsequently rescued into a preconditioned E3 medium (Figure 4 A and B). Buffered solution was pH 7.4 and it was administered to each well under treatment where larvae were, as indicated in Figure 4 B.

### Heart Rate Measurements

The heart rate was assessed on 8 and 10 dpf. Control and experimental zebrafish larvae were individually transferred to a depression slide with methylcellulose and placed under a binocular microscope. The heart rate was determined by counting the number of beats every 15 s and recorded as beats per minute (bpm) (see Video S1). Experiments were performed thrice on three larvae per group for each time point [27].

### Preparation of Histological Sections

For the fixation of samples, both treated and control animals were anesthetized by a tricaine methanesulfonate solution (MS-222, Sigma) at 0.3 g/l. Samples were then fixed by immersion in 4% v/v paraformaldehyde in PBS, pH 7.4 for 24 hours at 4°C. Following fixation, paraformaldehyde was removed with five washes of 5 minutes in PBS. Then, the samples were embedded in a mixture of agar 1.5% and sucrose (Panreac) 10% in PBS. Such mixture was heated and added to the plastic molds in which the animals were targeted. After the mixture was solidified, the larvae were cryoprotected in a 30% w/v sucrose solution in PBS for 24 h. Agar blocks containing cryoprotected larvae were frozen in a cryostat (Microm HM 560) and then cut at −28°C in 10-µm-thick parasagittal serial sections, which were collected on gelatinized slides and stored at −20°C until further use. We performed 55 histological sections and larvae were analyzed three times (n = 3) at 10 dpf [27].

### Hematoxylin-Eosin Staining

Histological sections were obtained as mentioned above and stained with hematoxylin-eosin to observe possible morphological changes. Briefly, the technique involves immersing the sections in eosin for 1 minute, then washing with water every 30 minutes and further incubating for 1 minute in hematoxylin. Finally, the samples were dehydrated in ethanol of increasing concentration for 5 minutes each, ending with three tanks of xylene, for 3 minutes each. The slides were mounted in Entellan (Merck KGaA, Darmstadt, Germany) for analysis and storage. Images of hematoxylin-eosin staining were taken in a light microscope (Olympus Provis AX70) coupled to a digital camera (DP70, Olympus).

Finally, to adjust the brightness and contrast to those observed directly under the microscope, Adobe ® Photoshop CS2 ® version 9.0 (Adobe Systems) was used [27].

### Immunohistochemistry in Tissue Sections

The sections were washed three times in PBS for 10 min to rehydrate and remove the agar. They were incubated for 1 h at room temperature (RT) in non-immune serum (Sigma) 5.0%, detergent Triton X-100 (Sigma) 0.2% and 1.0% DMSO in PBS. The serum used was made into the species of the secondary antibody.

Then, the primary antibodies were added and incubated for 24 hours at RT. After this incubation, the excess antibodies were removed with three washes with PBS and then the sections were incubated with the corresponding secondary antibodies conjugated with the appropriate fluorochrome for 1 h at RT. The secondary antibody was removed with three washes of 10 minutes each in PBS with fish gelatin 0.4% (Sigma-Aldrich).

In order to mark cell nuclei, tissue sections were incubated in 4′,6-diamidine-2-phenylindole (DAPI, Sigma) at a 1∶10,000 concentration for 7 minutes at RT, and then washed three times of 10 minutes each in PBS.

### Antibodies Used

-Polyclonal anti-calretinin (CalR) antibody 7696 (# 6B3 Swant, Bellinzona, Switzerland) at a 1∶10,000 concentration. This antibody has been widely used in the study of the neuroanatomy of teleosts, in adult animals as well as in embryos, larvae and juveniles [28], [29]. Secondary antibody labeled with Cy3 (red) [30].

-Anti-tyrosine hydroxylase (TH) antibody (Incstar, Stillwater, MN, USA) [31], at a 1∶1,000 concentration. Secondary antibody labeled with Cy2 (green).

The sections were examined under a microscope (Olympus Provis AX70) coupled to a digital camera (XM10, Olympus). The images were coded green (Cy2) and red (Cy3), giving yellow co-localization in merged images. The images were adjusted for brightness, contrast and colors using Adobe Photoshop 7.0 (Adobe Systems) [27].

### Statistical Analysis

Data were presented as mean ± standard deviation and analyzed by one-way analysis of variance (ANOVA) and Tukey's Multiple Comparison Test using GraphPad Prism v. 5. Only values with P<0.05 were accepted as significant.

## Results and Discussion

### Preparation of the DG4.5-Risp Complex

The methodology with the highest drug incorporation efficiency proved to be that related to the following parameters:

2.4×10^−3^ µmoles of DG4.5+0.24 µmoles of Risp (DG4.5:Risp 1∶100 mol:mol).
A chloroform:methanol 50∶50 v/v solution with HCl 0.1N added to a final pH of 3 (30 µl/ml incubation solution).
Film recomposition in 100 µl buffer PBS 2X.

Figure 5 shows the results obtained for the complexation of Risp with DG4.5, where 35 to 45 drug molecules per DG4.5 were incorporated.

The incubation solution percentage of solvents varied, and so the polarity changed. The greatest incorporation of Risp was obtained for the mixture chloroform:methanol 50∶50 v/v solution.

**Figure 6:**
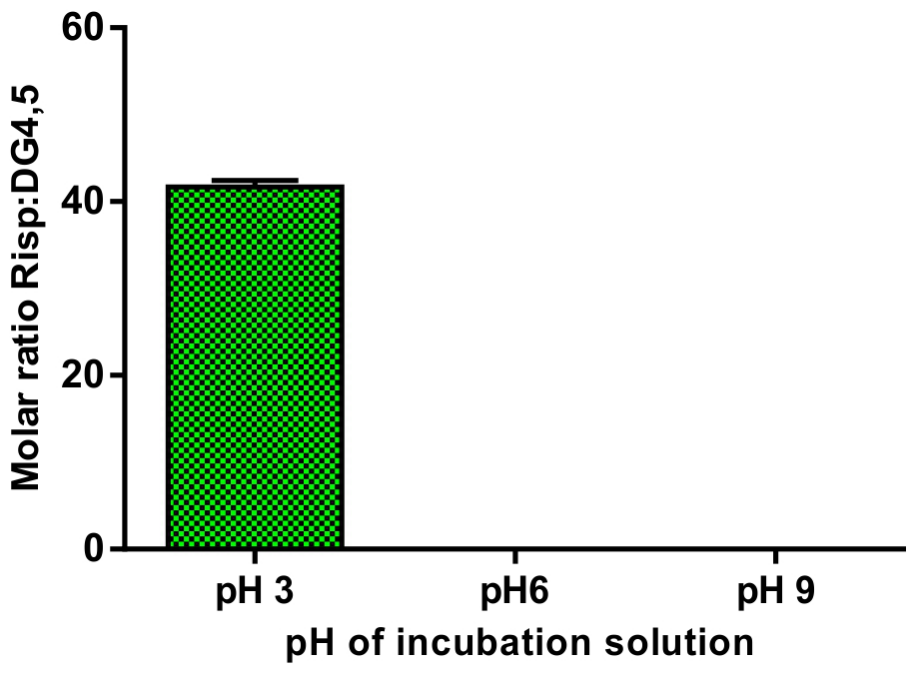
Optimization of incubation parameters. Moles of Risp per mole of DG4.5; data obtained from incubation solutions of chloroform:methanol 50∶50 v/v in different pH conditions.

Different pH conditions were then tested (pH 3–9) within the incubation solution in order to allow the highest incorporation of drug complexes in a 1∶100 molar ratio DG4.5:Risp and the incubation solvent composition of 50∶50 chloroform:methanol 50∶50 v/v solution. The solubility of the drug increased significantly at pH 3 (46 DG4.5-Risp molecules), proving that this is the optimum condition to obtain the DG4.5-Risp complex (Figure 6).

**Figure 7:**
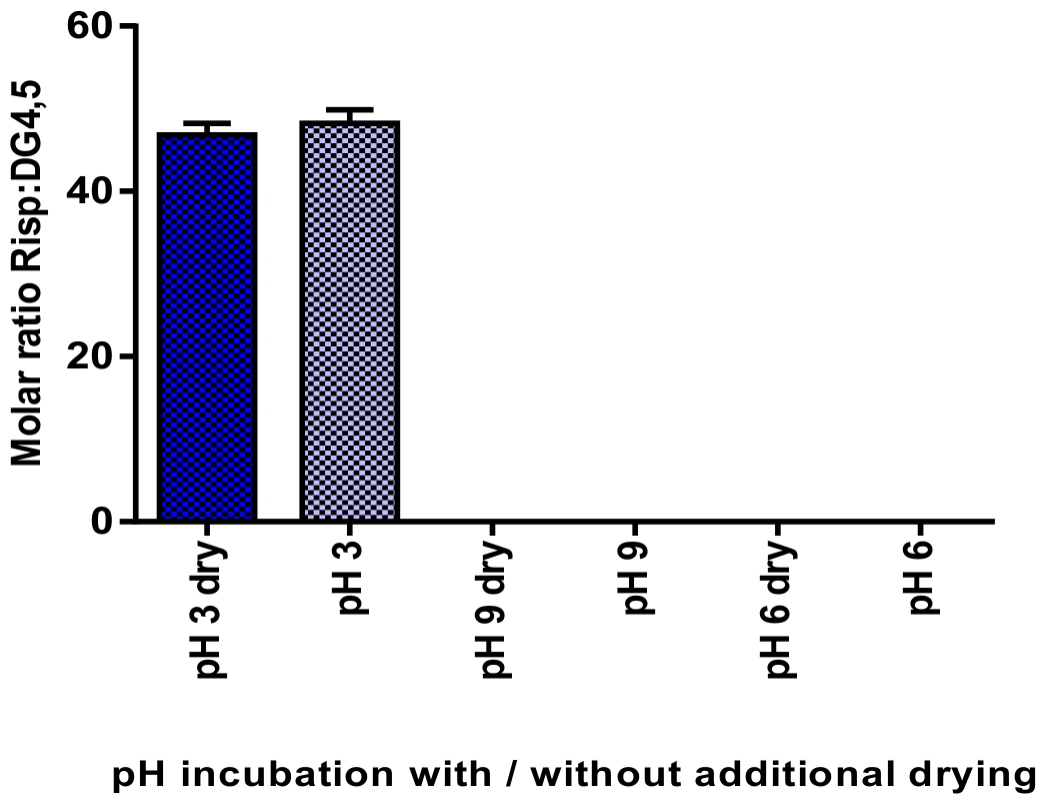
Moles of Risp per mole of DG4.5. Data from incubation with different chloroform:methanol 50∶50 v/v molar ratios solutions at different pHs, subjected or not to an additional drying process.

Finally, we analyzed whether, after the centrifuge vacuum drying operation, there were still remaining traces of organic solvents that could generate great variability in the amount of drug incorporated. No significant differences were observed between samples with and without the additional drying operation (Figure 7).

In a previous work where we examined the complexation of DG4 with Risp in different solvents, we found that in high ionic strength (1 M NaCl) few molecules of Risp were incorporated in each DG4 molecule. By simulation methodology, Welch and Muthukumar [32] determined that the density of dendritic profiles is suitable from that of dense core to that of a dense shell with salt concentration or pH modification. In addition, it has been reported that the nature of the intramolecular density profile and the position of the terminal groups are critical in utilizing dendrimers as drug hosts in controlled release systems [33], [34]. Ideally, to incorporate a drug, the branches of dendrimers should be highly extended. In the ionic strength tested in our previous work, this was not the case, and DG4 was not capable of incorporating a great number of drug molecules. This is consistent with data published by Ma et al. (2007) [34], who found that salt concentration is related to large changes in DG4 molecular conformation and decreased drug incorporation. The same trend was observed with solvent polarity [35]. It is known that the compact structures of the hydrophobic dendrimers presenting low accessibility to the hydrophobic pockets are favored by high polarity solvents [36].

For our particular system, i.e. hydrophilic dendrimers and Risp, we found that the best combination of solvents was chloroform:methanol 50∶50 v/v pH 3, which rendered 45 molecules of Risp per DG4.5. However, the 50∶50 condition presented minimal deviation. In this sense, methanol is necessary to stabilize the carboxyl-surface, but a non-polar solvent should also be present to improve the drug partitioning between the solvent and the inner hydrophobic pocket of the dendrimer, which will be wide open to incorporate the drug, but partially close to retain it. However, at 100% chloroform, no drug incorporation is achieved, since the drug partitions better in the highly non-polar solvent than in the hydrophobic DG4.5 pocket, and as the solvent is non-polar, DG4.5 compaction is expected and no drug entrapment in the hydrophobic DG4.5 inner can be achieved.

Finally, anionic DG4.5 is a weak acid capable of deprotonating the carboxyl ending of its branching points under physiological pH [37]. The branching points exhibit open conformations at low pH, due to the electrostatic repulsion between the superficial groups, which force branches to move away from each other. At pHs higher than 9, the branches come closer again as a consequence of the hydrogen bonds between the tertiary amines of the interior and the carboxyl ending group portioned of the surface, resulting in a compact structure [38]. Based on the results obtained in this work, we can conclude that the amount of Risp incorporated to DG4.5 is inversely proportional to pH values, which is also consistent with the literature.

### DG4.5-Risp Complex Stability

**Figure 8:**
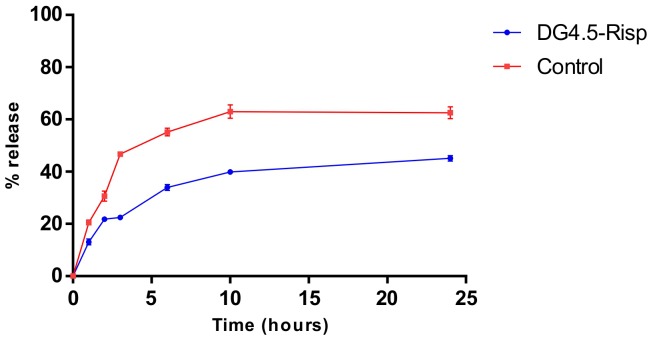
Release profile of the drug complexed to DG4.5. The graph indicates the percentage of drug released when found in the dendrimers or free form.

In contrast to that shown by free Risp, the release profile of the drug complexed with DG4.5 showed that the dendrimers functioned as nanocarriers (Figure 8). DG4.5-Risp complexes resulted in a 45.08% release in contrast to the 62.52% release of the free drug, after 24 h.

### Characterization of DG4.5-Risp Complexes

**Figure 9:**
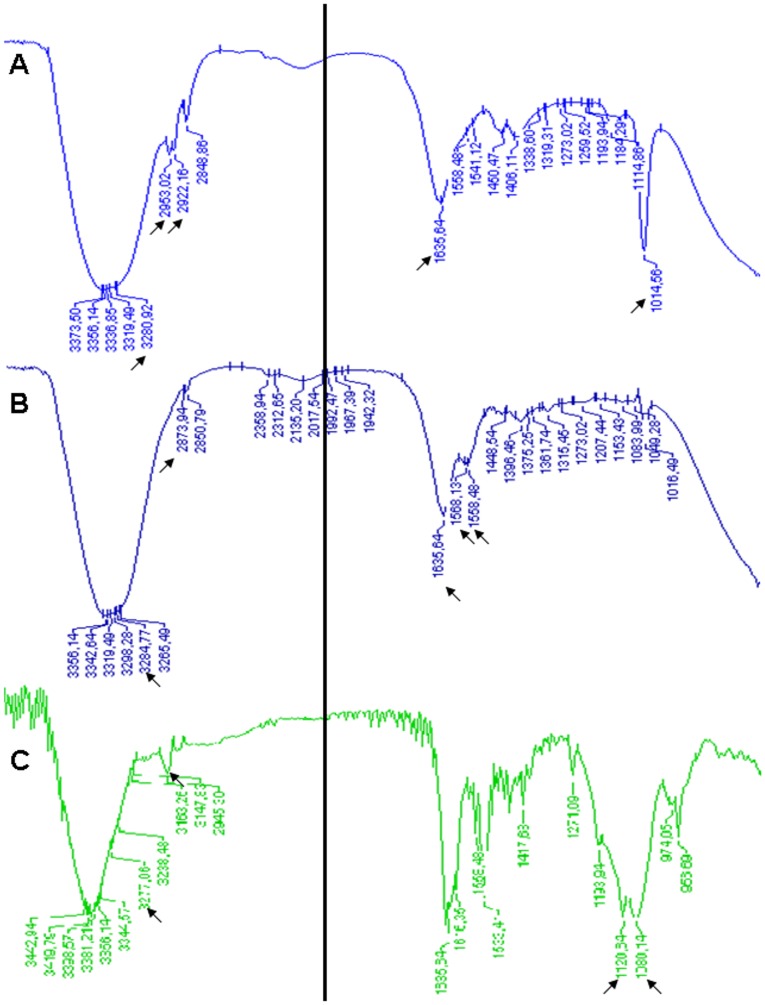
Drug interaction with the dendrimer. FTIR spectrum of Risp (A), DG4.5 (B) and DG4.5-Risp (C) solid state runs.

Figure 9 A shows the FTIR spectrum of the solid drug Risp. The bands observed correspond to the vibrational mode of the amide NH group at 3280 cm^−1^ and the corresponding bending at 1635 cm^−1^. The band of the aromatic ring movement around 1014 cm^−1^ is also present. The bands observed are also those related to stretching vibration of the CH_2_ group at 2922 and 2953 cm^−1^.

On the other hand, the FTIR spectrum of DG4.5 (Figure 9 B) showed an absorbance peak at 2873 cm−1, corresponding to the vibrational motion of the terminal carboxyl groups and a strong double band related to a symmetric vibrational motion of the carboxylate at 1568 cm−1. The spectrum also showed bands corresponding to the vibrational mode of the amide group NH at 3284 cm−1 and 1635 cm−1, corresponding to the flexion group. In addition, movements due to CH bonds, related to the dendrimer backbone chain within the core of DG4.5, were observed at 1558 cm−1.

The FTIR spectrum for the DG4.5-Risp complex (Figure 9 C) showed several differences when compared with the control spectrum. The most interesting change was that of the band shift of the -CH_2_ binding at 2945 cm^−1^, indicating the presence of hydrophobic interactions in the sample analyzed. We also observed a less pronounced shift of the amide band at 3277 cm^−1^, and several bands, especially at 1120 and 1080 cm^−1^, due to the stretching vibration of the carbonyl group CO bound, strongly indicating hydrophobic interactions between the dendrimers and the drug [7]. The resultant of dynamic light scattering of complexes wase the three indicated peaks of multimodal distribution, which diameters reached an average of 6.099 nm and 454.6 nm. The last peak indicates complex aggregates. Anionic dendrimers like DG4.5 often have zeta potentials more negative than −30 mV [39], we found that DG4.5-Risp complexes had a zeta potential of 5.83 mV, due to the conversion of the dendrimers' surface carboxyl groups with Risp molecules. This close to neutral charge could be responsible for the formation of aggregates [40]–[42].

### *In Vivo* Toxicity

Airhart et al. (2007) [43] exposed zebrafish embryos to seven different fluoxetine (a serotonin reuptake inhibitor) concentrations beginning at 10 hpf and up to 11 days post fertilization (dpf) to determine the lowest observable effective concentration (LOEC). In larvae exposed to 4.6 µM fluoxetine for 24-h intervals between 4 and 5 dpf, spontaneous swimming activity was significantly depressed compared to controls and remained depressed ones through 14 dpf. In addition, the core neuronal migration raphe to the spinal cord was observed between 3 and 6 dpf [43] and variations in the serotonin levels may affect the normal development of the central nervous system (CNS).

Based on the observations obtained in our previous work [27], we selected the concentrations to be used in the present work.

### Heart Rate Measurements

The effect of Risp exposure on circulation was qualitatively evaluated by observing the heart rate and blood flow through the ventral aorta-posterior cardinal vein channel in control versus treated larvae. These parameters provide an idea of the effects caused by free Risp and the DG4.5-Risp complex in early development stages and knowledge on the area of neuropharmacology. Here, we determined whether Risp or DG4.5-Risp affected blood circulation. To this end, 4 and 6 dpf larvae were exposed to 5 µM Risp or DG4.5-Risp for 24 h and their heart rate monitored at 8 and 9 dpf. Treated larvae exhibited normal heart rate as compared to controls (data not shown).

### Tissue Sections

#### i- Morphological Changes

**Figure 10:**
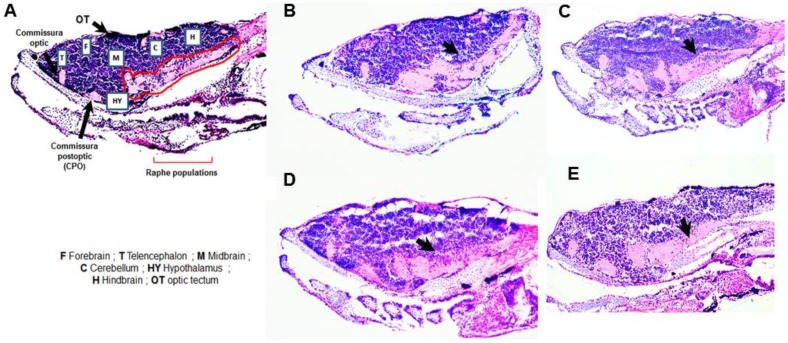
Images of histological sections of brain tissue stained with hematoxylin-eosin. A) control, B) risperidone (Risp) at 4 dpf, C) Risp at 6 dpf, D) DG4.5-Risp at 4 dpf, and E) DG4.5-Risp at 6 dpf. Larvae were analyzed three times (n = 3) at 10 dpf.

Both treated and control animals were fixed at 10 dpf, cut in serial sections and stained, as detailed in the Experimental Section. A 24-h exposure to risperidone or DG4.5-Risp on 4 dpf resulted in a larger area in the postoptic commissure and the raphe population zone and a cellular disorganization in the latter. This effect was observed in all treatments, but higher in animals treated with free Risp (Figure 10). Undoubtedly, the administration of this drug to animals at 4 dpf caused dramatic changes that persisted over time.

#### ii- Immunohistochemistry

For the histological analysis, crop images were obtained to include reference space area and optical angle for brain tissue. We used tyrosine hydroxylase (TH) to label dopaminergic neurons and calretinin (CalR) to label motoneurons.

**Figure 11:**
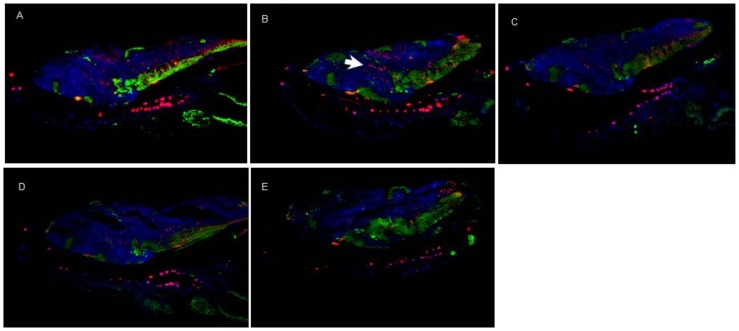
Immunohistochemistry images of brain tissue. Tyrosine hydroxylase, labeled with Cy2 (green), and Calretinin, labeled with Cy3 (red). A) control, B) risperidone (Risp) at 4 dpf, C) Risp at 6 dpf, D) DG4.5-Risp at 4 dpf, and E) DG4.5-Risp at 6 dpf. Larvae were analyzed three times (n = 3) at 10 dpf.

When the larvae were exposed to free Risp at 4 dpf, an increase in CalR-positive motoneurons was observed in the brain (Figure 11). The other treatments showed no changes in brain tissue with respect to controls (Figure 11).

**Figure 12:**
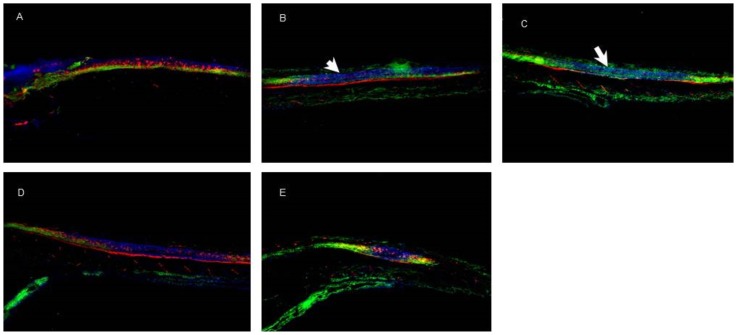
Immunohistochemistry images of spinal cord tissue. Tyrosine hydroxylase, labeled with Cy2 (green), and Calretinin, labeled with Cy3 (red). A) control, B) risperidone (Risp) at 4 dpf, C) Risp at 6 dpf, D) DG4.5-Risp at 4 dpf, and E) DG4.5-Risp at 6 dpf. Larvae were analyzed three times (n = 3) at 10 dpf.

The spinal cord showed a decrease in CalR-positive motoneurons in treatments with Risp alone (Figure 12). The other treatments showed no changes in brain tissue with respect to controls (Figure 12).

Several antipsychotic drugs produce a neurotoxic mechanism resulting from an increased or decreased concentration of serotonin both in the synaptic and extracellular spaces. In this sense, drug exposure at 4 or 5 dpf coincides with the initial appearance of raphe axons distributed throughout the entire length of the spinal cord in zebrafish [43]. Growth cones of these axons at 4 dpf were observed adjacent to reticulospinal neurons in the hindbrain and secondary motoneurons in the spinal cord. The temporal correlation between the growth of inferior raphe axons and growth cones throughout the spinal cord and the earliest morphological effects of antipsychotic drugs suggested that raphe axons were affected by the exposure to these drugs. However, the mechanism of toxicity by excess or deficit of serotonin was difficult to determine.

Antipsychotic drugs could alter extracellular levels of neurotransmitters and thereby modify the development of the CNS [43]–[46]. These changes suggest that the neuroanatomy is severely affected by exposure to free Risp but to a lesser extent than by DG4.5-Risp.

## Conclusions

Development of molecular nanostructures with well-defined particle sizes is of increasing interest in biomedical applications [6], [47]–[49]. Dendrimers, like other delivery systems, offer attractive properties that allow modifying the pharmacokinetics and bioavailability of drugs. These changes depend not only on the class of dendrimer, but also on the physicochemical nature of the complex that the dendrimer forms with the drug. Drugs can be complexed with dendrimers through encapsulation into void spaces (nanoscale container), association with the surface groups (nano-scaffolding), or both [6], [50]. The high density of surface groups (one amino group/nm^2^ for DG4) combined with the small size (4.5 nm diameter for the DG4 ellipsoids) result in a high area/volume ratio [51], [52], which can be modified controlling the environment ionic strength, pH, temperature, etc.

In summary, here we described the preparation, stability and characterization of the DG4.5-Risp complex. The best dendrimer-risperidone incorporation (46 risperidone molecules per dendrimer) was achieved with a mixture of chloroform:methanol 50∶50 v/v pH 3.

Then, we determined the *in vivo* effects of risperidone and DG4.5-Risp on the heart rate and brain development in zebrafish larvae. The most significant changes were observed when free risperidone was administered, but no changes were observed when larvae were treated with the complex. This could indicate a decrease in the side effects of the drug when administered as a complex, or a decrease in the effectiveness and/or arrival of the complex. Certainly, more studies are necessary to determine whether the complexed drug reaches the brain.

## Supporting Information

Video S1 **Heart Rate Measurements.** The heart rate was assessed on 8 and 10 dpf. Control and experimental zebrafish larvae were individually transferred to a depression slide with methylcellulose and placed under a binocular microscope. The heart rate was determined by counting the number of beats every 15 s and recorded as beats per minute.
(MP4)
